# Chk1 Activation Protects Rad9A from Degradation as Part of a Positive Feedback Loop during Checkpoint Signalling

**DOI:** 10.1371/journal.pone.0144434

**Published:** 2015-12-11

**Authors:** William F. Osorio-Zambrano, Scott Davey

**Affiliations:** 1 Division of Cancer Biology and Genetics, Queen’s University Cancer Research Institute, Kingston, Ontario K7L 3N6, Canada; 2 Department of Pathology and Molecular Medicine, Queen’s University, Kingston, Ontario K7L 3N6, Canada; 3 Department of Oncology, Queen’s University, Kingston, Ontario K7L 3N6, Canada; 4 Department of Biomedical and Molecular Sciences, Queen’s University, Kingston, Ontario K7L 3N6, Canada; St. Georges University of London, UNITED KINGDOM

## Abstract

Phosphorylation of Rad9A at S387 is critical for establishing a physical interaction with TopBP1, and to downstream activation of Chk1 for checkpoint activation. We have previously demonstrated a phosphorylation of Rad9A that occurs at late time points in cells exposed to genotoxic agents, which is eliminated by either Rad9A overexpression, or conversion of S387 to a non-phosphorylatable analogue. Based on this, we hypothesized that this late Rad9A phosphorylation is part of a feedback loop regulating the checkpoint. Here, we show that Rad9A is hyperphosphorylated and accumulates in cells exposed to bleomycin. Following the removal of bleomycin, Rad9A is polyubiquitinated, and Rad9A protein levels drop, indicating an active degradation process for Rad9A. Chk1 inhibition by UCN-01 or siRNA reduces Rad9A levels in cells synchronized in S-phase or exposed to DNA damage, indicating that Chk1 activation is required for Rad9A stabilization in S-phase and during checkpoint activation. Together, these results demonstrate a positive feedback loop involving Rad9A-dependend activation of Chk1, coupled with Chk1-dependent stabilization of Rad9A that is critical for checkpoint regulation.

## Introduction

The cell cycle activates different checkpoints after DNA damage to ensure that DNA repair is completed before the continuation of cell cycle progression. The S-phase checkpoint is especially critical because it ensures that DNA replication is accurate, thereby maintaining genome stability. Cell cycle regulation pathways are composed of signals, sensors of the signal, mediators, transducers and effectors proteins [1,2]. The Rad9A sensor phosphoprotein plays a role in regulating several cell cycle checkpoints, including regulation of Chk1 activation in S-phase and G2/M checkpoints [3–6].

The PCNA-like 9-1-1 complex is a trimer composed of Rad9A, Rad1, and Hus1, which is loaded onto DNA by Rad17–RFC complex [7–12]. Rad9A is phosphorylated on multiple sites in normal cycling cells and rapidly hyperphosphorylated and loaded onto DNA after DNA damage [13–18]. Rad9A interacts with TopBP1 through phosphorylations on S387 and S341 [19,20]. Rad9A helps to position TopBP1 next to ATR-ATRIP complex for ATR activation via TopBP1 activation domain [19–21]. An activated ATR phosphorylates Chk1 on S317 and S345 [22,23]. Brca1 ubiquitinates and stabilizes Claspin for Chk1 activation [24–28]. An activated Chk1 phosphorylates Cdc25A [29,30]. SCFβ-TrCP ubiquitin ligase recognizes a phosphorylated Cdc25A, resulting in Cdc25A ubiquitination and degradation preventing Cdk2 dephosphorylation and cell cycle progression [31]. In addition to helping position TopBP1 next to ATR, Rad9A is also involved in the nuclear localization of Claspin [32].

Rad9A hyperphosphorylation after DNA damage is observed at different time points [13,15]. ATM rapidly phosphorylates Rad9A on S272 after ionizing radiation (IR) exposure [13,15], and a late phosphorylation of Rad9A is detected after genotoxic stress [15]. Rad9A late phosphorylation after damage requires prior phosphorylation on S387, and is not observed under conditions of Rad9A overexpression [15]. Thus, Rad9A late phosphorylation after damage seems to require prior activation of Rad9A-TopBp1-ATR-Chk1 pathway. The preferred phosphorylation consensus sequence for ATR is SQ [33], and Rad9A has a unique SQ consensus at S272, which becomes phosphorylated early in the damage response [34,35]. A kinase candidate for the late phosphorylation of Rad9A after DNA damage is Chk1, which leads to the possibility of a positive feedback mechanism for Rad9A stabilization to increase Chk1 activation in checkpoint maintenance. We present evidence here supporting the presence of a positive feedback loop between Chk1 and Rad9A.

## Materials and Methods

### Cell culture

HeLa Tet-Off cells were established according to the manufacturer’s instructions as described previously [14]. HeLa Tet-Off cells were cultured in Dulbecco’s modified Eagle’s medium (Sigma-Aldrich, Oakville, Canada) with 10% fetal bovine serum (Invitrogen, Burlington, Canada) in a humidified environment at 37°C and 5% CO_2_. The human retinal pigment epithelial cells that stably expresses the human telomerase reverse transcriptase subunit (hTERT-RPE1, CCL‐28) from the ATCC cell repository (Manassas, VA) were maintained as above with Dulbecco’s modified Eagle’s medium/F-12 medium (Sigma-Aldrich, Oakville, Canada) and 10% fetal bovine serum (Invitrogen).

### Cell synchronization

In order to obtain HeLa Tet-Off or hTERT-RPE1 cell populations enriched in S-phase, 1 x 10^6^ cells were seeded the day before onto each 100-mm plate, and then, synchronized in G1/S border with a single 18 h thymidine block (2 mM). Then, cells were washed once with phosphate-buffered saline (PBS) and release for 2 h in fresh media for treatment in S-phase.

### Drug treatments and irradiation

The DNA damage agent bleomycin sulfate (Bioshop, Burlington, Canada) was dissolved in sterile saline (9g/L NaCl) at a stock concentration of 10 mg/ml. Cells were treated with bleomycin (BLEO) at ~ 50% confluence. The Chk1 inhibitor UCN-01 (Sigma-Aldrich, Oakville, Canada) was dissolved in DMSO at a stock concentration of 1 mM and further diluted at a final concentration of 300 nM in complete media. Cells were treated with 300 nM UCN-01 or solvent (DMSO). Cycloheximide (CHX), Ready-Made Solution (Sigma-Aldrich, Oakville, Canada) is a 100 mg/ml CHX solution in DMSO (C4859) that was further diluted at a working concentration of 100 μg/ml in complete media. Cells were exposed to 100 μg/ml CHX or DMSO. MG132, Ready-Made Solution (Sigma-Aldrich, Oakville, Canada) is a 10 mM MG132 solution in DMSO (M7449) that was further diluted at a working concentration of 10 μM in complete media. Cells were treated with 10 μM MG132 or solvent. Cells were exposed to 10 Gy of IR using a Victoreen Electometer ^137^Cs γ-irradiator at a dose rate of 0.45Gy/min (Atomic Energy of Canada, Mississauga, ON).

### Antibodies

The antibodies used for immunoblotting were Purified Mouse Anti-hRad9A (611324) from BD Transduction Laboratories (Mississauga, Canada); Rad9 Antibody (M-389): sc-8324, Chk1 (FL-476): sc-7898, and Cdc25C Antibody (C-20): sc-327 from Santa Cruz Biotechnology (Santa Cruz, CA, USA); Phospho-Chk1- Ser317 (2344) from Cell Signalling (Whitby, Canada); Anti-GAPDH antibody (ab15822), Anti-Ubiquitinin antibody (ab7780), Anti-Chk1 antibody [AF7H4] (ab80615), Anti-Rad1 antibody (ab76830), Anti-HUS1 antibody (ab96297) and Anti-Cdc25C (phospho S216) antibody [E190] (ab32051) from Abcam (Toronto, Canada), and also, Phospho-Rad9-S272 Antibody (AP3232a) from ABGENT (San Diego, CA, USA). Rad9A immunoprecipitation was performed using polyclonal chicken antibodies raised against Rad9A as described previously [7].

### Immunoprecipitations and Immunoblotting

Cell harvesting was performed by washing once with 5 ml of PBS, adding 2 ml of 2X trypsin for 5 min at 37°C, and collecting the cells with 5 ml of PBS. Cells were lysed in NETN buffer [10% glycerol, 120 mM NaCl, 20 mM Tris-HCl (pH 8.0) and 0.5% NP40] supplemented with 2X Halt™ Protease Inhibitor Cocktail-EDTA-Free (Thermo Fisher Scientific, Ottawa, Canada), 416 mM β- glycerophosphate, 5 mM CaCl2, 25 mM MgCl2 and 100 U DNase I per ml of cell lysate (Thermo Fisher Scientific, Ottawa, Canada). Cells were lysed for 20 min at room temperature with softly mixing by inverting each tube every 5 min. At the end of the incubation, EDTA was added at a final concentration of 1 mM to stop DNase activity. Cell lysates were centrifuged at 16,000 X *g* for 15 min at 4°C. Soluble cell lysates were normalized for total protein, an equal volume of 3X SDS-PAGE sample buffer was added, and then, heated at 95°C for 5 min. Samples were resolved on an 8% SDS-PAGE acrylamide gel, and then, transferred to a Hybond nitrocellulose Membrane (G.E. Healthcare, Mississauga, Canada) using a Criterion™ Blotter cell (an electrophoretic transfer cell from Bio-Rad) for 18 h at 19 V. Membranes were blocked with 5% non-fat milk powder (Bioshop, Burlington, Ontario) or 5% bovine serum albumin (Bioshop, Burlington, Ontario) in PBS/ 0.1% TWEEN 20 (PBST) for 30 min at room temperature (RT) on a rocking platform. Blocked membranes were incubated overnight with primary antibodies at 4°C on a rocking platform. The next day, membranes were briefly washed three times with PBST, and three times more for 5 min each at RT on a rocking platform. Membranes were incubated with HRP-conjugated secondary antibodies in PBST for 1 h at RT on a rocking platform. Immunoblots were briefly washed three times with PBST for 5 min each at RT on a rocking platform. Membranes were incubated for 5 min at RT with SuperSignal West Pico Chemiluminescent Substrate (Thermo Fisher Scientific Inc., Ottawa, Canada). The membrane chemiluminescence signal was detected by exposure to UltraCruz™ Autoradiography Film (Santa Cruz Biotechnology, Santa Cruz, CA, USA). Alternatively, the membrane chemiluminescence signal was detected using a Kodak image station 4000mM Pro (Rochester, NY, USA). Densitometry analysis was performed using images obtained from a Kodak image station 4000mM Pro and the Carestream Molecular Imaging Software, Version 5.0 (Carestream Molecular Imaging, New Haven, CT). In the case of Rad9A immunoprecipitation, soluble supernatants were precleared with 100 μl of PrecipHen® (Aves Labs, Tigard, OR) per ml of cell lysate for 30 min at 4°C on an orbital nutator. A total of 7 μg of affinity purified chicken polyclonal antibodies directed against Rad9A were added per 1 ml of the precleared cell lysates and incubated overnight on a Nutator Mixer at 4°C. Immunocomplexes (1 ml) were immunoprecipitated with 100 μl of PrecipHen® slurry (Aves Labs) for 6 hours on a Nutator Mixer at 4°C. Beads were washed once with cold NETN buffer and twice with cold PBS. Proteins were eluted from the beads with 100 μl of 1.5X SDS-PAGE sample buffer at 95°C for 5 min, and then, beads were spun to recover the eluted proteins. Chk1 immunoprecipitation was performed with rabbit polyclonal anti Chk1 antibody (sc-7898) from Santa Cruz Biotechnology (Santa Cruz, CA, USA). Chk1 immunoprecipitation was performed using 2 μg of rabbit anti Chk1 antibody per mL of precleared cell lysate. Immunocomplexes were immunoprecipitated with 100 μl of 50% slurry of protein A sepharose (Biovision, Milpitas, CA). Preclearing, incubation times, washes and elution were similar to the process used for Rad9A immunoprecipitation.

In the case of immunoprecipitation in denaturing conditions cells were lysed in denaturing buffer [1% SDS, 20 mM Tris-HCl (pH 8.0), 10% glycerol, 1 mM DTT, 15 mM N-ethylmaleimide, 416 mM β- glycerophosphate and 2X Halt™ Protease Inhibitor Cocktail-EDTA-Free (Thermo Fisher Scientific)]. Then, cell extracts were denatured by boiling for 10 min at 95°C and sonicated. Cell lysates were centrifuged at 16,000 X *g* for 10 min at room temperature, the supernatants were collected, and then, diluted with 9 vol of modified RIPA buffer [20 mM Tris-HCl (pH 8.0), 0.5 mM EDTA, 150 mM NaCl, 0.5% Igepal CA-630, 10% glycerol, 10 mM N-Ethylmaleimide, 0.5% BSA, 416 mM β- glycerophosphate and 2X Halt™ Protease Inhibitor Cocktail-EDTA-Free (Thermo Fisher Scientific)]. Soluble cell lysates were normalized for total protein and immunoprecipitations were carried out as mention above.

### Flow Cytometry

A number of 1 x 10^6^ HeLa Tet-Off cells were fixed overnight in 70% ethanol and 30% PBS with 1% fetal bovine serum (FBS) at -20°C. Then, cells were washed twice with cold PBS, resuspended in 1 ml of PBS, 1% FBS, and 0.5 mg/ml RNase-A (Bioshop, Burlington, Ontario), and treated for 40 min at 37°C. Cells were collected by centrifugation, resuspended in 0.5 ml of PBS, 0.5 μg/ml propidium iodide (PI) and 0.1 mg/ml RNase A. Samples were incubated for 1 h and analyzed using a flow cytometer (Beckman Coulter EPICS ALTRA HSS). In the case of Dual BrdU-PI staining, Cell Proliferation Labeling Reagent (GE Healthcare Life Sciences, Baie d’Urfe, QC) was added 1 hour before harvesting in a dilution 1:1000. Cells were fixed and treated with RNase-A as described before. On the following day, cells were washed once with cold PBS and incubated in 1 ml of 2N HCl and 0.5% Triton X-100 (DNA denaturation solution) for 30 min at room temperature. Then, cells were subjected to centrifugation to remove supernatant and resuspended in 2 ml of neutralization buffer (0.1M sodium borate, pH 8.5) for 30 minutes at room temperature. Cells were washed once with 3 ml PBS with 0.5% TWEEN 20 and resuspended with 200 μl of PBS with 0.5% TWEEN 20. Anti-BrdU FITC (eBioscience, San Diego, CA) was added (1 μg) to the cells for 1 h at room temperature. Finally, cells were washed with 3 ml of cold PBS and resuspended in 0.5 ml of PBS, 0.5 μg/ml propidium iodide (PI) and 0.1 mg/ml RNase A to be incubated for 1 h at 4°C before flow cytometry analysis.

### Short interfering RNA (siRNA) transfections

Two validated Chk1 siRNAs were used to study the effects of Chk1 inhibition on Rad9A stabilization and accumulation. Chk1 siRNA AM51331 (C1) with the target sequence 5’-GGAGAGAAGGCAAUAUCCAtt-3’ and Chk1 siRNA AM51333 (C2) with the target sequence 5’-GGGAUAUUAAACCAGAAAAtt-3’ from Life Technologies (Burlington, ON). As a negative control siRNA was used AM4611 (N) with the target sequence 5’-AGUACUGCUUACGAUACGGTT-3’ from Life Technologies (Burlington, ON). Chk1 and the negative control siRNA transfections were performed using Lipofectamine 2000 (Life Technologies) following manufacturer’s recommendations. Briefly, 1 x 10^5^ cells were seeded per well (6-well plates) the day before of transfection. Cells were transfected with 5 μl of Lipofectamine 2000 and siRNA at a final concentration of 100 nM. Transfected cells were synchronized (S-phase) 48 h later.

## Results

### DNA damage increases Rad9A phosphorylation and accumulation

**Fig 1:**
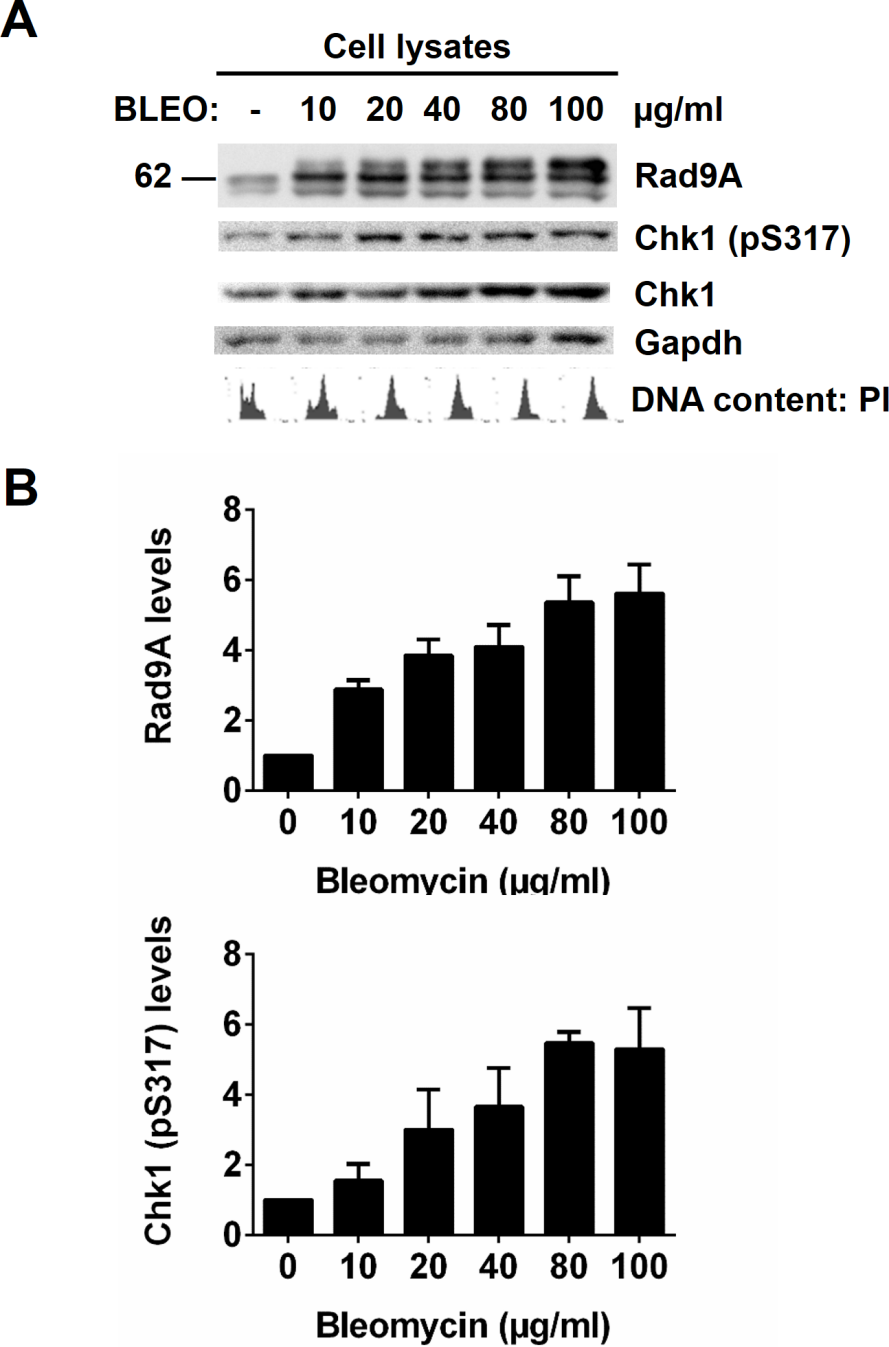
Rad9A is accumulated and hyperphosphorylated in cells exposed to bleomycin for 20 h. Synchronized HeLa Tet-Off cells (G1/S border) were released for 2 h, and then, cells were exposed (+) or not (-) to different concentrations of BLEO (10, 20, 40, 80 or 100 μg/ml) and harvested 20 h later. (A) Cell lysates were immunoblotted with antibodies directed against the proteins indicated, and a representative immunoblot is presented. Cell cycle analysis was determined by DNA content with propidium iodide (PI). (B) Rad9A (all Rad9A bands detected in the immunoblot were included in the densitometry analysis) and Chk1 (pS317) relative levels are expressed as normalized values of optical density using GAPDH as the loading control. All error bars represent the standard error of the mean (SEM) from three independent experiments.

Rad9A shows a late phosphorylation after DNA damage in S-phase, and this phosphorylation requires the prior phosphorylation of Rad9A on S387 [14,15]. Rad9A phosphorylation on S387 allows Rad9A and TopBp1 interaction for Rad9A-TopBp1-ATR-Chk1 pathway activation [19–21]. One interpretation of this result is that Chk1 activation is required for Rad9A late phosphorylation after damage to allow Rad9A stabilization, acting as a feedback mechanism for checkpoint maintenance. To determine if Rad9A is hyperphosphorylated and accumulated after DNA damage in S-phase, HeLa Tet-Off cells were synchronized to S-phase, then treated with increasing concentrations of the DNA-damaging agent bleomycin (BLEO) for 20 h. Rad9A is phosphorylated on multiple sites during a normal cell cycle, and DNA damage increases Rad9A phosphorylation [13,15,16]. An increased Rad9A phosphorylation after DNA damage produces Rad9A slow migrating forms when cell lysates are subjected to SDS-PAGE and immunoblot analysis [14,15]. In agreement with previous studies, a Rad9A slower migration band was detected with BLEO treatment and this hyperphosphorylated band became more abundant with increasing concentrations of BLEO (Fig 1A). In addition to Rad9A hyperphosphorylation, we determined that Rad9A levels were increased with BLEO concentration in cells exposed for 20 h (Fig 1A and 1B). To determine Chk1 activation levels in this context, a phospho-specific antibody directed against a phosphorylated serine 317 was used [36–38], and results showed that Chk1 activation (level of pS317) was increased with increasing BLEO concentration (Fig 1A and 1B). These results indicate that prolonged exposure to a radiomimetic agent such as BLEO produces an accumulation of hyperphosphorylated Rad9A forms as well as an increase in Chk1 activation.

### Release from bleomycin exposure reduces Rad9A levels

**Fig 2:**
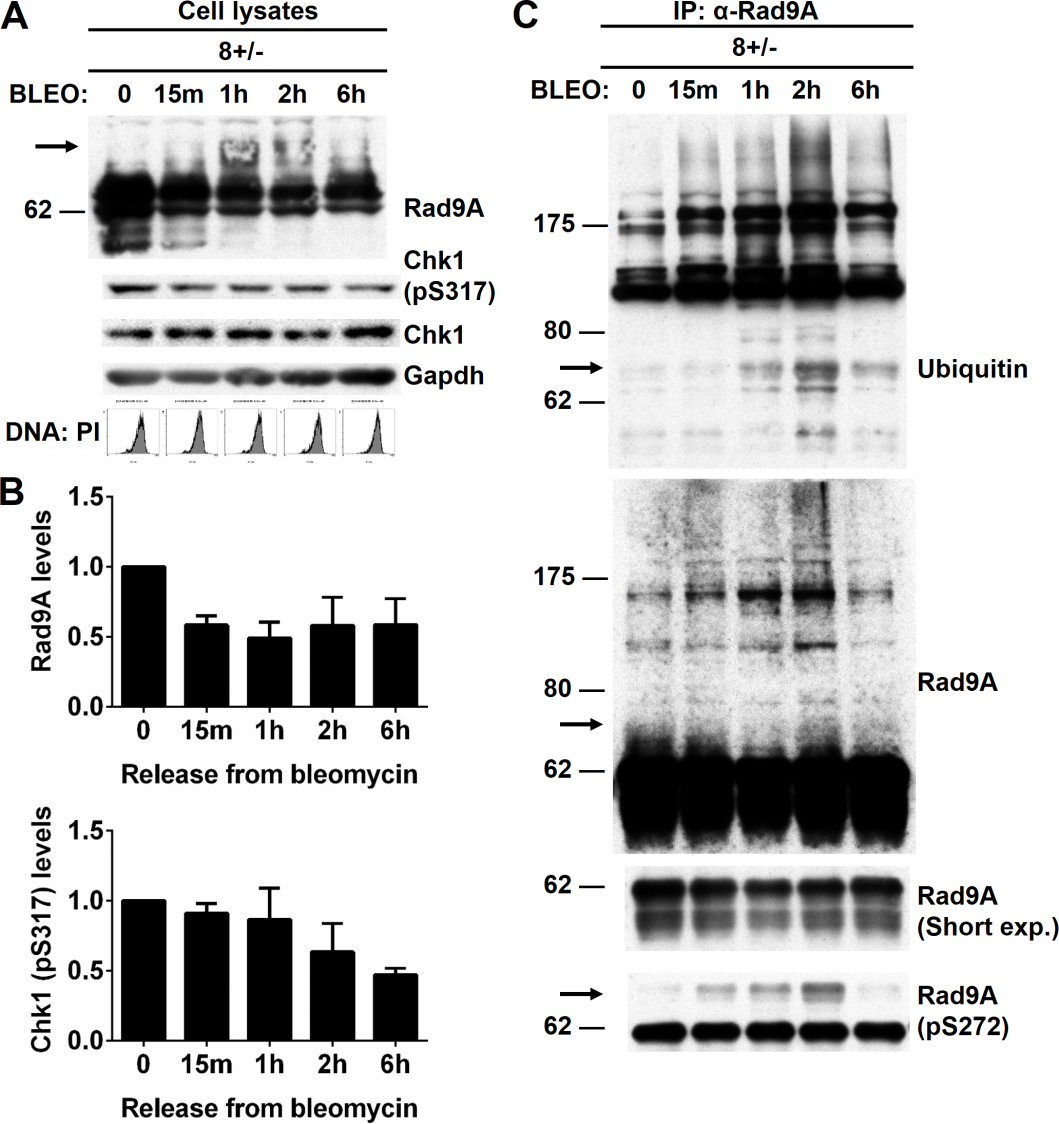
Rad9A is polyubiquitinated, and Rad9A levels are reduced when cells are released from bleomycin treatment. Synchronized HeLa Tet-Off cells in S-phase were exposed to 100 μg/ml BLEO for 8 h and harvested (8+/-0) or exposed for 8 h, released for 15 min, 1, 2 or 6 h, and harvested (8+/-15m, 1h, 2h, or 6h). (A) Cell lysates were immunoblotted with antibodies directed against the proteins indicated, and a representative immunoblot is presented. Cell cycle analysis was determined by DNA content with PI. (B) Rad9A (all Rad9A bands detected in the immunoblot were included in the densitometry analysis) and Chk1 (pS317) relative levels are expressed as normalized values of optical density using GAPDH as the loading control. All error bars represent SEM from three independent experiments. (C) Cell lysates were immunoprecipitated (IP) with antibodies directed against Rad9A. Then, immunoprecipitated proteins were immunoblotted with antibodies directed against the proteins indicated. Arrows around 70 KDa indicate an increased ubiquitination of phosphorylated Rad9A on S272.

A long exposure to BLEO increases Rad9A accumulation and Chk1 activation (Fig 1A and 1B). Mailand *et al*. (2006) showed that during checkpoint recovery, Plk1 phosphorylation of Claspin mediates Claspin and SCFβ-TrCP ubiquitin ligase interaction, leading to Claspin degradation and preventing Chk1 reactivation [26]. Since BLEO exposure in S-phase leads to Rad9A accumulation, we hypothesized that removing the source of damage would lead to a reduction of Rad9A levels during checkpoint recovery. To test this, synchronized HeLa Tet-Off cells were treated with BLEO for 8 h or exposed for 8 h and released for 15 min, 1, 2 or 6 h. Bleomycin treatment for 8 h produced Rad9A accumulation, and removing BLEO from the cells rapidly decreased Rad9A levels (Fig 2A and 2B). Chk1 activation also decreased after BLEO removal (Fig 2A and 2B). These results together show that Rad9A accumulation and increased Chk1 activation observed after DNA damage are attenuated with the removal of the genotoxic agent.

### Rad9A polyubiquitination is prevented after DNA damage

We found that Rad9A levels quickly decreased after BLEO removal suggesting protein degradation (Fig 2A and 2B). We hypothesized that Rad9A degradation could be mediated by the ubiquitin–proteasome pathway as part of a checkpoint recovery process [26,39,40]. To study this possibility, HeLa Tet-Off cells (S-phase) were exposed to BLEO or exposed to BLEO and released for different periods of time (15 min, 1, 2 or 6 h). Cell lysates were then immunoprecipitated (IP) under nondenaturing conditions with antibodies directed against Rad9A. Rad9A immunoprecipitates immunoblotted with antibodies directed against ubiquitin, Rad9A or Rad9A phosphorylation on S272 showed an increased Rad9A ubiquitination with reduced Rad9A levels when cells were released from BLEO (Fig 2C). Rad9A is rapidly phosphorylated by ATM after DNA damage [13,15]. It was interesting to observe an increased ubiquitination of phosphorylated Rad9A on S272 when BLEO was removed (see arrows around 70 kDa) (Fig 2C).

**Fig 3:**
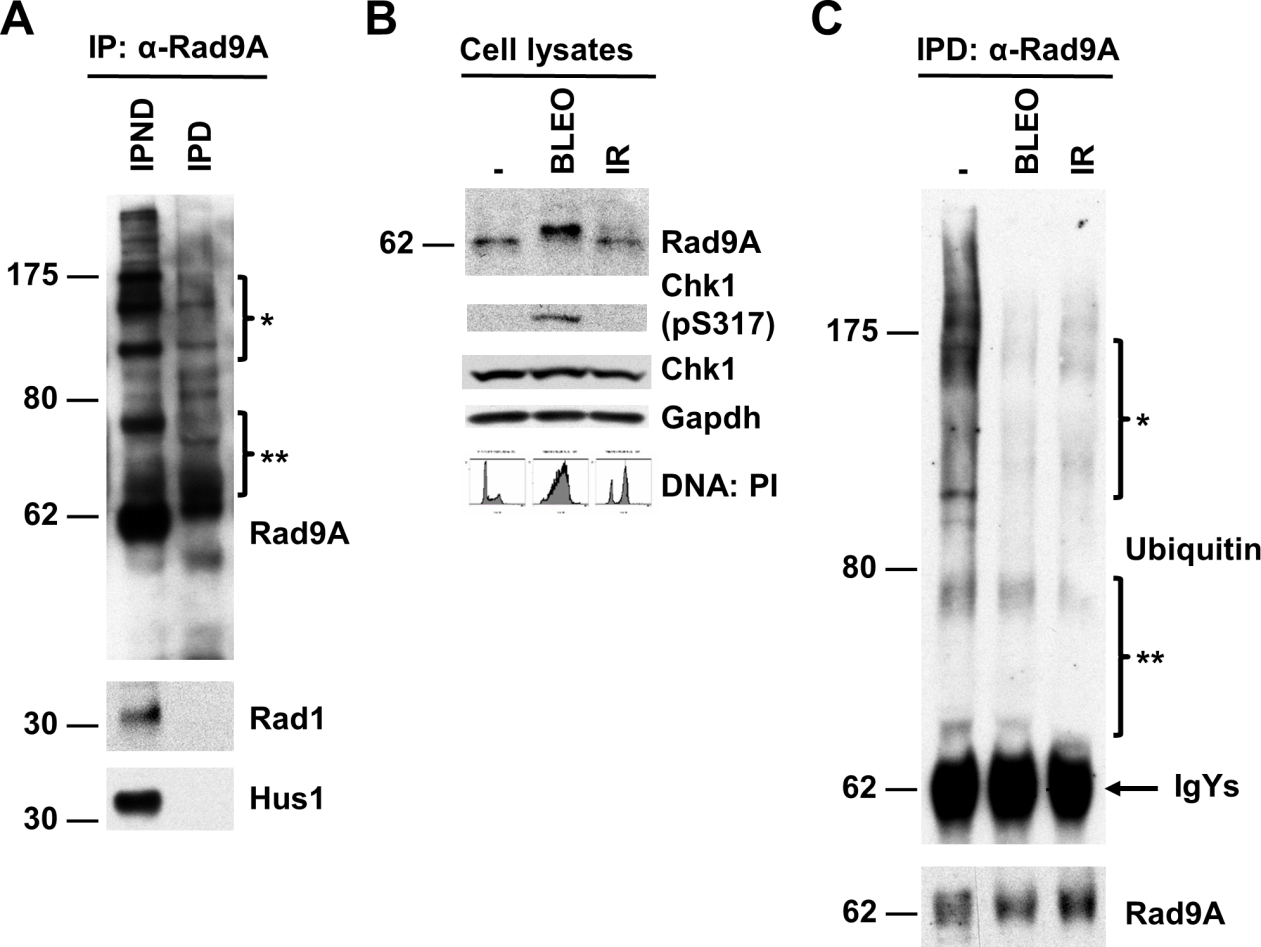
DNA damage reduces Rad9A polyubiquitination. (A) Rad9A was immunoprecipitated from cell lysates of asynchronous HeLa Tet-off cells in non-denaturing conditions (IPND) or in denaturing conditions (IPD). Then, Rad9A immunoprecipitates were immunoblotted with antibodies directed against the proteins indicated. (B) Asynchronous HeLa Tet-off cells were exposed or not to 100 μg/ml BLEO or 10 Gy of IR and harvested 20 h later. Cell lysates were immunoblotted with antibodies directed against the proteins indicated. Cell cycle analysis was determined by DNA content with PI. (C) Cell lysates from the samples mentioned above were prepared in denaturing conditions and immunoprecipitated (IPD) with antibodies directed against Rad9A, and then, immunoblotted with antibodies directed against the proteins indicated. Bracketed regions (* and **) indicate corresponding areas of overlap between protein bands detected with anti-ubiquitin and anti-Rad9A antibodies.

Since Rad9A associates tightly with Rad1 and Hus1 to form the 9-1-1 complex [7–9,41,42], we wanted to be sure that the ubiquitination we observed in Rad9A immunoprecipitations was specific to Rad9A. To do this, Rad9A immunoprecipitations were performed after a stringent denaturing treatment. Rad9A co-immunoprecipitation with Hus1 or Rad1 (IPND) was prevented when Rad9A was immunoprecipitated under denaturing conditions (IPD) (Fig 3A). To confirm that Rad9A is polyubiquitinated in cycling cells, and DNA damage prevents its polyubiquitination, asynchronous HeLa Tet-Off cells were exposed or not to BLEO or ionizing radiation (10 Gy) and harvested 20 h later (Fig 3B). Then, Rad9A was immunoprecipitated under denaturing conditions (IPD). Rad9A polyubiquitination was observed in cyclin cells, and this polyubiquitination was reduced after DNA damage (Fig 3C). All these results together show that DNA damage prevents Rad9A polyubiquitination.

### Chk1 inhibition increases Rad9A polyubiquitination and reduces Rad9A levels

**Fig 4:**
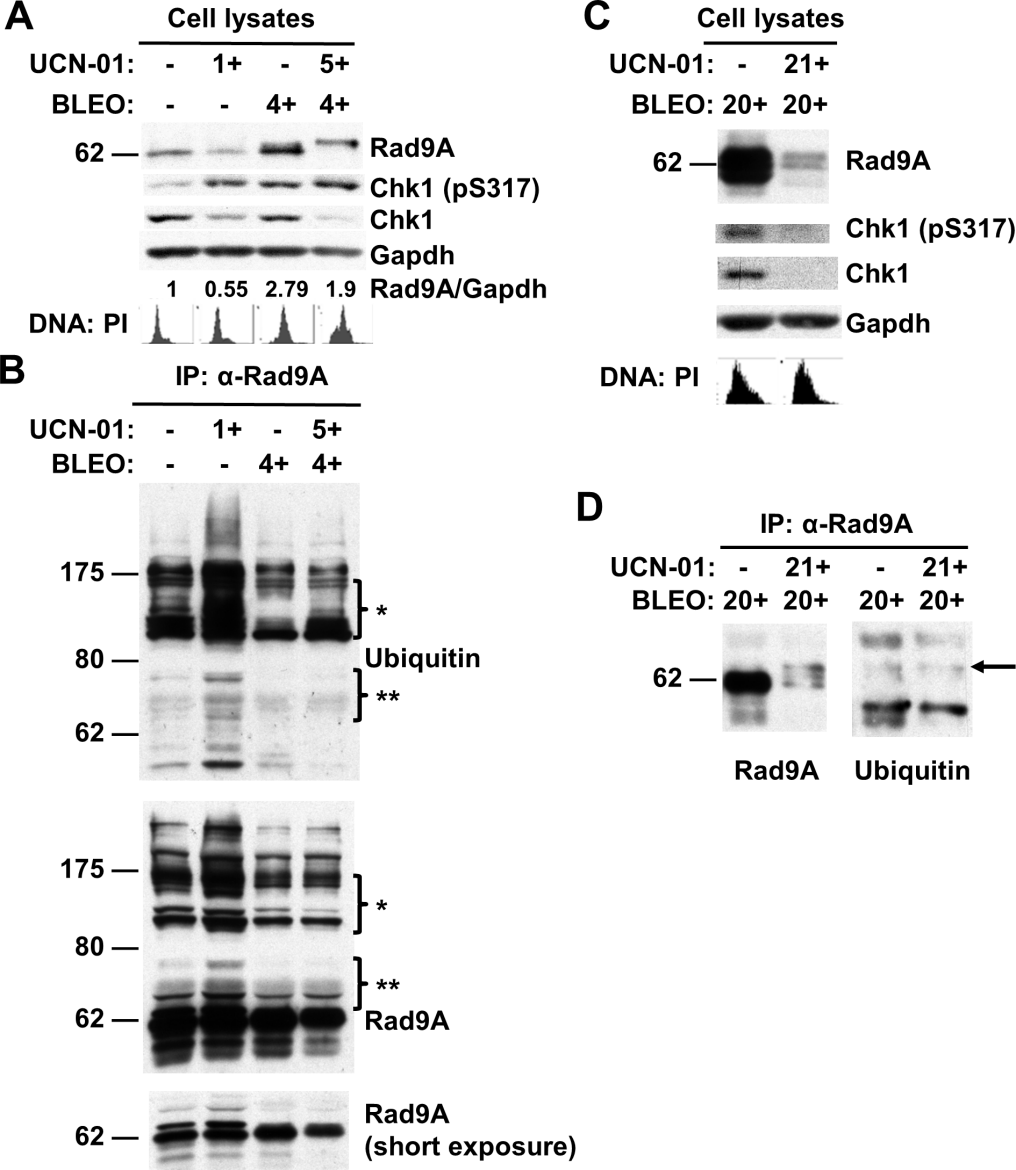
UCN-01 treatment reduces Rad9A levels and increases Rad9A polyubiquitination. (A) Synchronized HeLa Tet-off cells (G1/S-phase border) were released for 1 h and exposed or not to 300 nM UCN-01 (1 h) and harvested. Synchronized HeLa Tet-off cells (G1/S-phase border) were released for 2h and exposed to 100 μg/ml BLEO (4 h) or a combination of both (4h) and harvested (UCN-01 or solvent were added 1 h before BLEO). Cell lysates were immunoblotted with antibodies directed against the proteins indicated. Rad9A relative levels are expressed as normalized values of optical density using GAPDH as the loading control. Cell cycle analysis was determined by DNA content with PI. (B) Cell lysates from the samples mentioned above were immunoprecipitated with antibodies directed against Rad9A. Then, immunoprecipitated proteins were immunoblotted with antibodies directed against the proteins indicated. Bracketed regions (* and **) indicate corresponding areas of overlap between protein bands detected with anti-ubiquitin and anti-Rad9A antibodies. (C) Synchronized HeLa Tet-off cells (G1/S border) were released for 2 h and exposed to 100 μg/ml BLEO (20 h) or in combination with 300 nM UCN-01 (20 h) and harvested. UCN-01 was added 1 h before BLEO. Cell lysates were immunoblotted as previously described. Cell cycle analysis was determined by DNA content with PI. (D) Cell lysates from (C) were immunoprecipitated and immunoblotted as previously described.

We observed that prolonged BLEO exposure increases Chk1 activation and Rad9A accumulation (Fig 1A and 1B), and release from BLEO treatment reduces Chk1 activation and Rad9A levels (Fig 2A and 2B). Chk1 activation is required for Claspin stabilization [43]. Since, Rad9A stabilization seems to correlate with Chk1 activation, and Claspin stabilization requires Chk1 activation, we wanted to determine if Rad9A stabilization was also dependent on Chk1 activation. Sørensen *et al*. (2004) determined that during physiological S-phase, the ATR-Chk1-Cdc25A pathway is activated in a ‘surveillance mode’ to limit the rate of replication origin firing [44]. To determine if Chk1 is required for Rad9A stabilization in synchronized cells in S-phase, we used the specific Chk1 inhibitor, UCN-01 [45,46]. Synchronized HeLa cells (G1/S border) were released for 1 h, and then, treated (1+/-) or not (-/-) with UCN-01 and harvested 1 h later. Reduced Rad9A levels were observed when UCN-01 was added for 1h in early S-phase (Fig 4A). We next wanted to determine if Rad9A polyubiquitination increases when Chk1 is inhibited with UCN-01 in S-phase. Cell lysates of the samples mentioned above were immunoprecipitated (IP) in nondenaturing conditions with antibodies against Rad9A. Rad9A showed increased Rad9A polyubiquitination when Chk1 is inhibited by UCN-01 in S-phase (Fig 4B). These results indicate that Chk1 activation is required to maintain Rad9A steady-state levels and prevent an increased Rad9A polyubiquitination in S-phase. Given that Rad9A was accumulated after BLEO exposure (Fig 1A and 1B), we decided to study the effects of Chk1 inhibition with UCN-01 in Rad9A accumulation in case of DNA damage. To study the effect of UCN-01 treatment on Rad9A levels in cells exposed to DNA damage, synchronized HeLa Tet-Off cells (S-phase) were treated with BLEO for 4 h alone or in combination with UCN-01 (UCN-01 was added one hour before BLEO treatment). Rad9A showed a slower migration with reduced Rad9A levels when UCN-01 is added before BLEO treatment (Fig 4A). We also observed an increased Rad9A polyubiquitination from the immunoblotting of Rad9A immunoprecipitates when Chk1 is inhibited before BLEO treatment (IP was performed under nondenaturing conditions) (Fig 4B). It was observed an increased Chk1 phosphorylation on S317 with reduced Chk1 levels when UCN-01 was added to the cells (1 or 5 h) which agrees with previous reports that show increased phosphorylation of ATR substrates when Chk1 is inhibited [47–49]. These results together show that prior Chk1 inhibition prevents Rad9A accumulation when cells are exposed to DNA damage. Short exposure to UCN-01 (1 or 5 h) increased Rad9A polyubiquitination and reduced Rad9A levels in cells exposed or not to DNA damage in S-phase (Fig 4A and 4B). To determine if previous Chk1 inhibition could also prevent Rad9A accumulation during prolonged BLEO exposure (20 h), synchronized HeLa Tet-Off cells (S-phase) were treated with BLEO for 20 h alone or in combination with UCN-01 (UCN-01 was added 1 h before BLEO treatment). Rad9A, Chk1 or Chk1 activation (pS317) showed a significant reduction in their levels when Chk1 is inhibited before a prolonged BLEO treatment (Fig 4C). Rad9A immunoprecipitates (IP was performed under nondenaturing conditions) immunoblotted with antibodies directed against Rad9A or ubiquitin showed increased Rad9A ubiquitination with combined treatment of UCN-01 and BLEO for 20 h (Fig 4D, see arrow). Altogether, these results show that previous Chk1 inhibition prevents Rad9A stabilization in S-phase, and also, its accumulation when cells are exposed to DNA damage.

### Chk1 inhibition with small interfering RNA (siRNA) reduces Rad9A stabilization and accumulation

**Fig 5:**
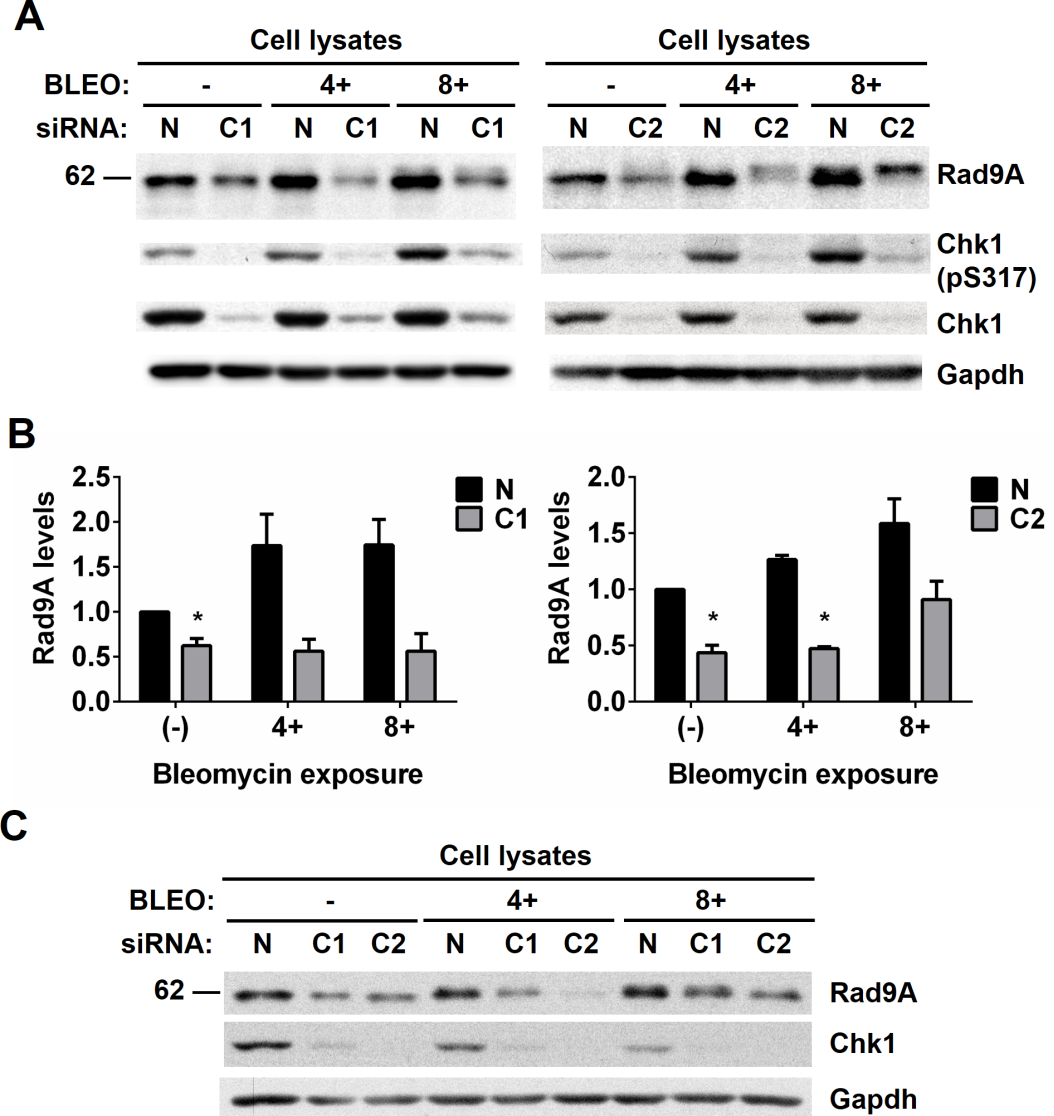
Reduction of Chk1 levels with Chk1 siRNA reduces Rad9A stability and accumulation after DNA damage. HeLa Tet-Off cells were transfected with non-silence control (N) or a siRNA against Chk1 (C1 or C2). Then, cells were synchronized in S-phase and harvested (-), exposed to 100 μg/ml BLEO for 4 h (4+) and harvested or exposed to 100 μg/ml BLEO for 8 h (8+) and harvested. (A) Cell lysates were immunoblotted with antibodies directed against the proteins indicated, and a representative immunoblot is presented. (B) Rad9A (all Rad9A bands detected in the immunoblot were included in the densitometry analysis) relative levels are expressed as normalized values of optical density using GAPDH as the loading control. All error bars represent SEM from three independent experiments. *P < 0.05. (C) hTERT-RPE1 cells were transfected with non-silence control (N) or a siRNA against Chk1 (C1 or C2), and then, treated as mention above. Cell lysates were immunoblotted with antibodies directed against the proteins indicated.

We determined that Chk1 inhibition with UCN-01 prevents Rad9A stabilization and accumulation after DNA damage (Fig 4). UCN-01 is considered a specific inhibitor of Chk1 [45,46] but it has been demonstrated that it also inhibits protein kinase Cδ (PKCδ) [50], a kinase that phosphorylates Rad9A as part of the apoptotic response to DNA damage [51]. To confirm that Chk1 is required for Rad9A stabilization in S-phase and accumulation after DNA damage, Chk1 protein levels were reduced with Chk1 small interfering RNA (siRNA) in HeLa Tet-Off cells, and then, the synchronized cells (S-phase) were exposed or not to BLEO (4 or 8 h). We found that Rad9A levels were reduced in S-phase when Chk1 levels are reduced (Fig 5A and 5B), and also, a reduction in Chk1 levels prevented Rad9A accumulation after BLEO exposure (Fig 5A and 5B). We also observed that Chk1 depletion with siRNA produced slower migrating forms of Rad9A when cells are exposed to BLEO (Fig 5A), and these slow migrating forms were similar to the ones observed with combined treatment of UCN-01 and BLEO (Fig 4A).

To determine whether the stabilization of Rad9A by Chk1 was specific to HeLa Tet-Off cells, we repeated this experiment in the untransformed hTERT-RPE1 cell line that stably express the human telomerase reverse transcriptase subunit (Fig 5C). As it was observed in Fig 5A, Rad9A levels were reduced with Chk1 siRNA in hTERT-RPE1 cells exposed or not to BLEO (Fig 5C). All these results together confirm that Chk1 is required for Rad9A stabilization and accumulation following DNA damage in both HeLa Tet-Off cells and hTERT-RPE1 cells.

### Rad9A and Chk1 coimmunoprecipitate during S-phase

**Fig 6:**
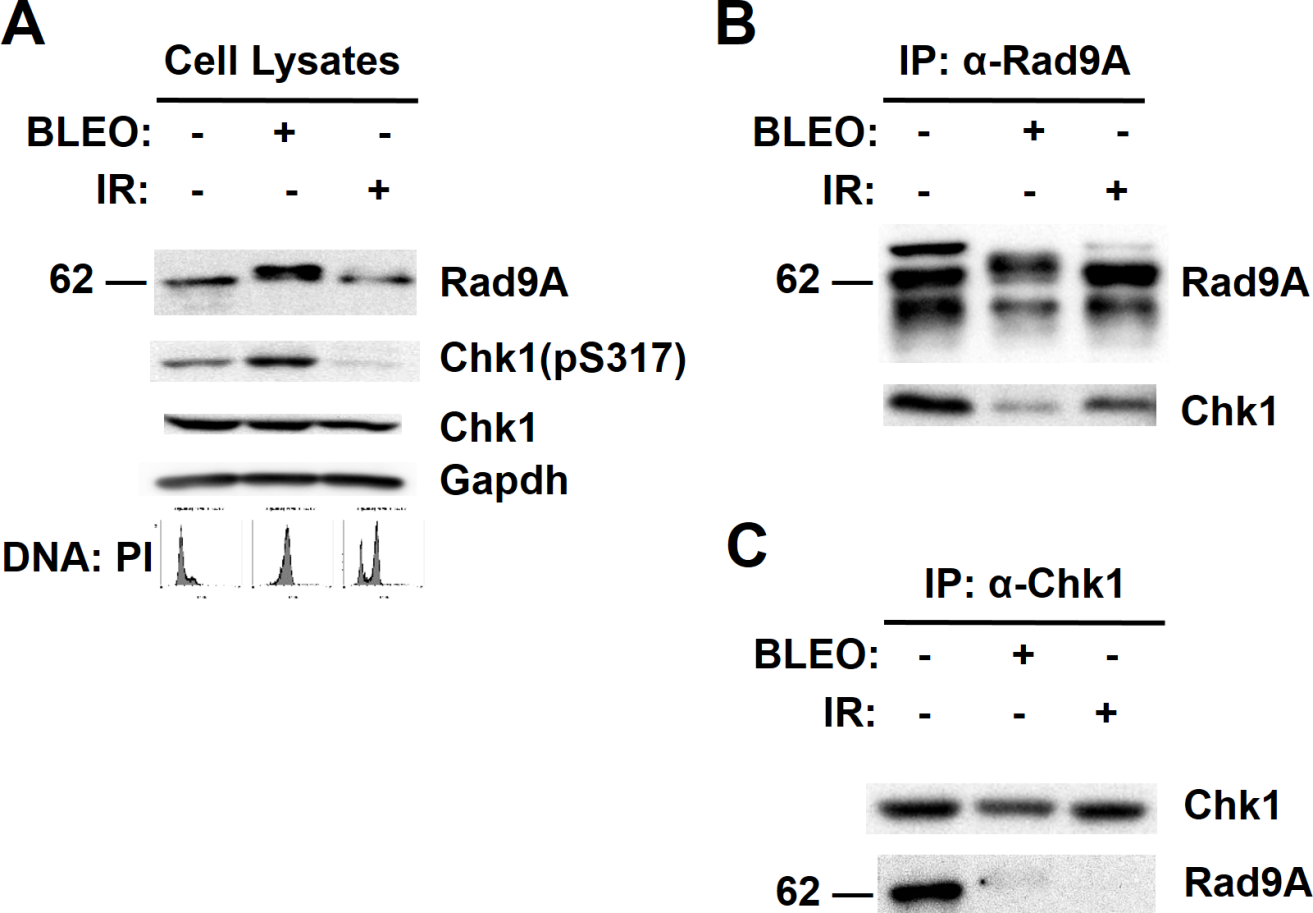
Rad9A coimmunoprecipitates with Chk1 during S-phase. Synchronized HeLa Tet-Off cells (S-phase) were harvested, exposed to 100 μg/ml BLEO for 20 h and harvested, or exposed to 10 Gy of IR and harvested 20 h later. (A) Cell lysates were immunoblotted with antibodies directed against the proteins indicated, and a representative immunoblot is presented. (B) Cell lysates were immunoprecipitated (IP) with antibodies directed against Rad9A. Then, immunoprecipitated proteins were immunoblotted with antibodies directed against the proteins indicated. (C) Cell lysates were immunoprecipitated (IP) with antibodies directed against Chk1. Then, immunoprecipitated proteins were immunoblotted with antibodies directed against the proteins indicated.

Since Chk1 is required for Rad9A stabilization, we next examined the possibility of an interaction between Rad9A and Chk1. To determine if Rad9A coimmunoprecipitates with Chk1, synchronized HeLa Tet-Off cells (S-phase) were harvested, treated with BLEO (20 h and harvested) or exposed to IR (harvested 20 h later) (Fig 6A). Cell lysates were then immunoprecipitated (IP) under nondenaturing conditions with antibodies directed against Rad9A or Chk1. Rad9A immunoprecipitates immunoblotted with antibodies directed against Chk1 showed Chk1 co-immunoprecipitation (CO-IP) under all the conditions with the highest CO-IP observed in cells enriched in S-phase (Fig 6B). In the case of Chk1 immunoprecipitation, we observed Rad9A CO-IP in cells synchronized in S-phase, and very weak or not detectable CO-IP in cells exposed to BLEO or recovering from IR exposure (Fig 6C). These results together show that Rad9A and Chk1 coimmunoprecipitate in both directions in S-phase, and this coimmunoprecipitation is reduced after prolonged exposed to BLEO or at the moment of DNA damage recovery (IR exposure recovery).

### Rad9A degradation is not mediated by the proteasome system

**Fig 7:**
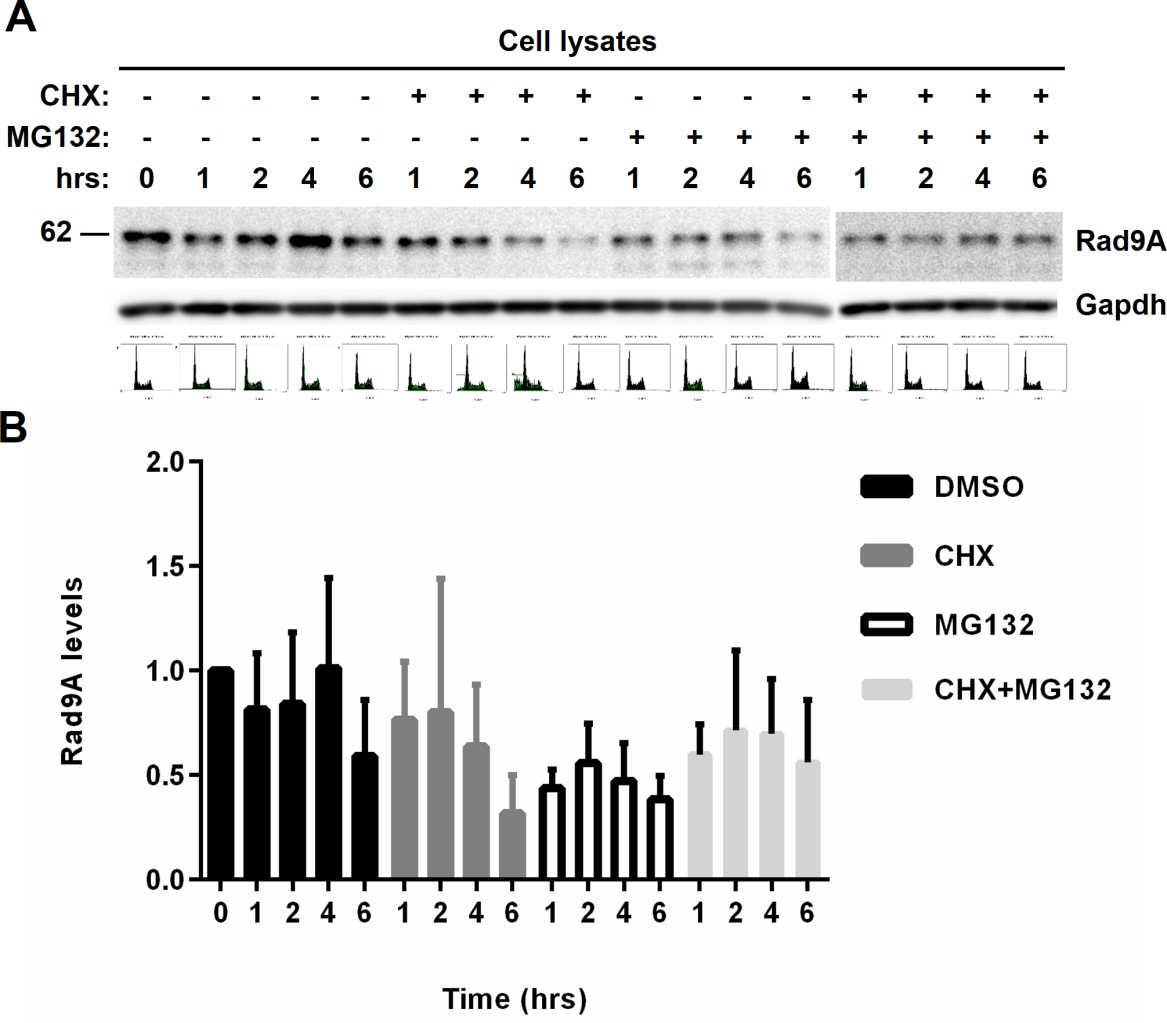
Exposure to MG132 increases Rad9A degradation. (A) Asynchronous HeLa Tet-Off cells were exposed or not to 100 μg/ml cycloheximide (CHX), 10 μM MG132 or a combination of both for the indicated periods of time and harvested. Cell lysates were immunoblotted with antibodies directed against the proteins indicated. (B) Rad9A (all Rad9A bands detected in the immunoblot were included in the densitometry analysis) relative levels are expressed as normalized values of optical density using GAPDH as the loading control. All error bars represent SEM from three independent experiments.

We determined that Rad9A accumulates after DNA damage, and this accumulation is dependent on Chk1 (Figs 1, 3 and 4). We also observed that recovery from DNA damage and Chk1 inhibition with UCN-01 reduces Rad9A levels and increases its polyubiquitination (Figs 2 and 3). Most of the intracellular proteins are degraded by the ubiquitin proteasome pathway (UPP) [52] and proteins involved in Chk1 activation like Brca1 and Claspin are degraded by the UPP [43,53]. We wondered about the possibility of Rad9A degradation by the UPP at the moment of checkpoint recovery. The antibiotic Cycloheximide (CHX) inhibits protein synthesis [54], and MG132 is a proteasome inhibitor that prevents the degradation of ubiquitinated proteins [55]. To determine if Rad9A is degraded by the UPP, asynchronous HeLa Tet-Off cells were exposed or not to cycloheximide (CHX), MG132 or a combination of both for different lengths of time. Rad9A degradation was observed after 4 h of CHX treatment (Fig 7A and 7B). Surprisingly, MG132 treatment reduced Rad9A levels after 1h (Fig 7A and 7B), and a combination of CHX and MG132 showed Rad9A degradation levels similar to CHX treatment (7A and B). These results indicate that Rad9A degradation is mediated by an active mechanism, and surprisingly, the inhibition of the ubiquitin-proteasome dependent pathway with MG132 increases Rad9A degradation.

**Fig 8:**
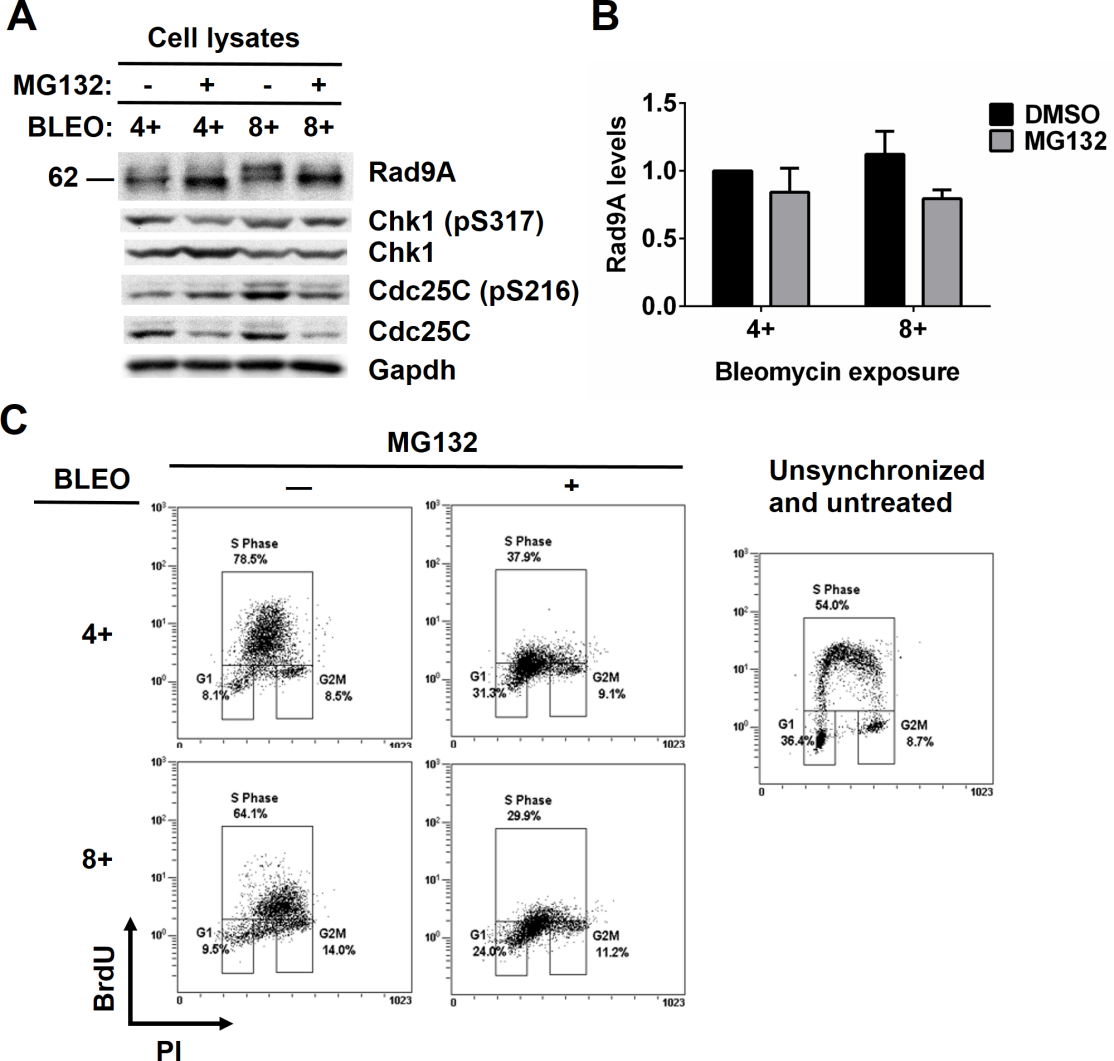
Exposure to MG132 reduces Rad9A phosphorylation and impairs checkpoint activation in cells exposed to bleomycin. Synchronized HeLa Tet-Off cells (G1/S border) were exposed for 2 h to DMSO or 10 μM MG132. Then, 100 μg/ml BLEO was added to the cells for 4 h (4+) and harvested or 8 h (8+) and harvested. (A) Cell lysates were immunoblotted with antibodies directed against the proteins indicated, and a representative immunoblot is presented. (B) Rad9A (all Rad9A bands detected in the immunoblot were included in the densitometry analysis) relative levels are expressed as normalized values of optical density using GAPDH as the loading control. All error bars represent SEM from three independent experiments. (C) The cell cycle of cells from (A) was determined by labeling and staining the cells with BrdU, staining total DNA with PI, and performing flow cytometry analysis.

Jacquemont *et al*. (2007) showed that the proteasome inhibitor, MG132, impairs the checkpoint activation that is observed after IR exposure [56]. Since, we found that Chk1 is required for Rad9A stabilization and accumulation (Figs 4 and 5), we wondered if the reduction of Rad9A levels produced by MG132 in asynchronous cells was connected with an effect on Chk1 activation (Fig 7A and 7B). To determine the effect of MG132 on Rad9A levels in case of DNA damage, synchronized HeLa Tet-Off cells (G1/S border) were exposed to DMSO or MG132 for 2 h, and then, BLEO was added for 4 or 8 h. A reduction in Rad9A phosphorylation was observed with the combined treatment of MG132 and BLEO (4 or 8 h) with an accumulation of Rad9A around 62 kDa (Fig 8A). It was also observed a reduction in Chk1 phosphorylation (S317), Cdc25C phosphorylation (S216) and Cdc25C levels in combined treatment of MG132 and BLEO after 8 h (Fig 8A). A small reduction of Rad9A levels was observed when MG132 was added before BLEO (Fig 8B). BrdU-PI dual staining analysis showed a reduction of cells in S-phase and an increased number of cells in G1-phase (Fig 8C). These results are consistent with previous results that report checkpoint impairment in cells treated with MG132 and then exposed to IR [56]. It was also observed a reduced incorporation of the proliferation agent BrdU when MG132 was added before BLEO (Fig 8C). All these results together show that Rad9A active degradation process is not mediated by the proteasome system.

## Discussion

**Fig 9:**
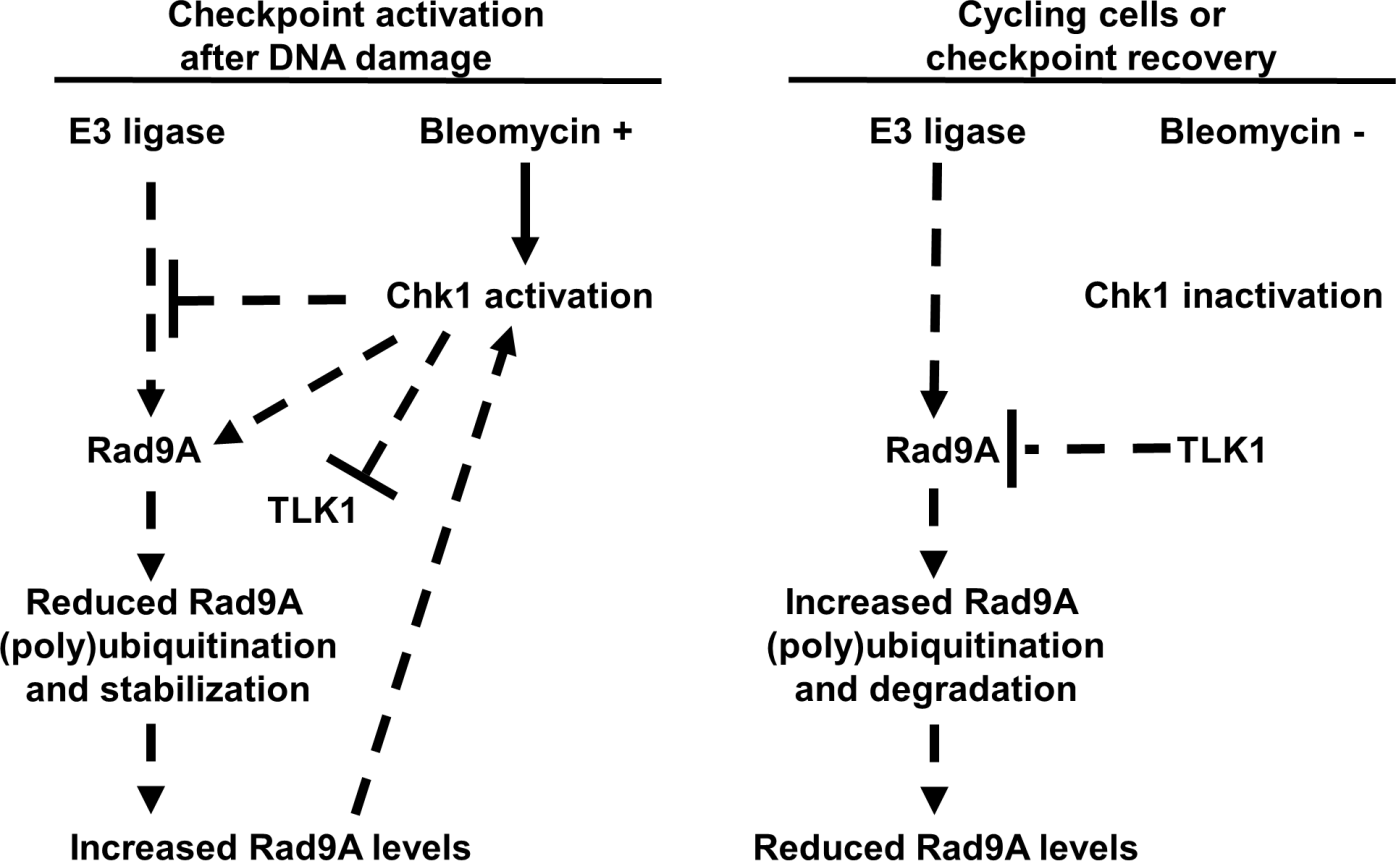
Rad9A stabilization model. In the case of DNA damage, an activated Chk1 phosphorylates Rad9A or other proteins (TLK1) as a feedback mechanism to prevent Rad9A (poly) ubiquitination and degradation. Then, increased Rad9A levels will increase and maintain Chk1 activation allowing checkpoint establishment. In the case of checkpoint recovery, a reduced Chk1 activation will allow Rad9A polyubiquitination and degradation with further Chk1 inactivation.

In this work, we have identified a positive feedback loop between Chk1 and Rad9A. This positive feedback loop allows Rad9A accumulation and increased Chk1 activation for checkpoint maintenance during prolonged exposure to DNA damage. The elucidation of the details of how Chk1 protects Rad9A from degradation will help to understand the different processes that ensure checkpoint maintenance in case of DNA damage. Based on these findings, we propose a model in which after DNA damage, an activated Chk1 prevents Rad9A (poly) ubiquitination and degradation to allow Rad9A accumulation and increased Chk1 activation for checkpoint maintenance. Chk1 could stabilize Rad9A directly by phosphorylating it or indirectly by phosphorylating another protein or proteins to prevent Rad9A destabilization. In the case of cycling cells or checkpoint recovery, the lack of Chk1 activation or Chk1 deactivation will allow Rad9A (poly) ubiquitination and degradation (Fig 9).

Rad9A phosphorylation is required for the activation of S-phase, G2/M and G2 decatenation checkpoints [6,13–15,19]. Here, we have linked Rad9A hyperphosphorylation after DNA damage to both Rad9A accumulation and Chk1 activation (pS317) (Fig 1). Furthermore, we have shown that Rad9A is polyubiquitinated in cycling cells, and this polyubiquitination is reduced after DNA damage (Fig 3C). On the other hand, we have demonstrated that Rad9A is polyubiquitinated with a reduction in its protein levels following BLEO removal (Fig 2). We have also demonstrated reduced Chk1 activation following the removal of BLEO (Fig 2A and 2B). Since Rad9A accumulates at checkpoint activation (Fig 1), a reduction in Rad9A levels could act as a mechanism for exit from the checkpoint. In the case of checkpoint recovery, others have already demonstrated that Claspin is phosphorylated by Plk1 to promote Claspin interaction with βTrCP ubiquitin ligase, leading to Claspin degradation and subsequent Chk1 inactivation [26]. Here, we have shown that Rad9A polyubiquitination and reduced Rad9A protein levels are observed during recovery from the checkpoint (Fig 2) indicating that Rad9A ubiquitination and degradation is another process that leads to Chk1 inactivation, and allows exit from the checkpoint.

Positive and negative feedback loops have been observed at some points in the cell cycle and checkpoint control [57,58]. An active, Cyclin B-Cdk1 complex phosphorylates Cdc25 phosphatases inducing Cdc25C activation [59], Cdc25A stabilization [60] and Cdc25B nuclear retention [61] as a positive feedback loop to dephosphorylate Cdk1 on T14 and Y15 for further activation of Cyclin B-Cdk1 complex. The cyclin B-Cdk1 complex is also part of a negative feedback loop to inactivate the kinases Wee1 [62,63] and Myt1 [64,65] that phosphorylate Cyclin B-Cdk1 complex on T14 and Y15. In the case of checkpoint activation, it has been observed that Chk1 is required for Claspin stabilization [43], and Claspin is necessary for Chk1 activation [24–27,43] suggesting a positive feedback loop for checkpoint maintenance. We showed that bleomycin exposure increases Rad9A levels and Chk1 activation (Fig 1), and BLEO removal reduces both Rad9A levels and Chk1 activation (Fig 2). Rad9A late damage phosphorylation seems to be dependent on previous Chk1 activation [14, 13].

We demonstrated that Chk1 activation is required to prevent Rad9A polyubiquitination and degradation (Fig 4). We have also demonstrated that reducing Chk1 levels affects Rad9A stability and prevents its accumulation in transformed and untransformed cells exposed or not to DNA damage (Fig 5). Since Rad9A is required for Chk1 activation [3–5] and Chk1 activation is required for Rad9A accumulation at checkpoint maintenance (Fig 4), we propose that Rad9A and Chk1 are part of a positive feedback loop for checkpoint maintenance. Chk1 inactivation leads to catastrophic mitotic death [66,67]. Sustained Chk1 activation is required for checkpoint maintenance after DNA damage to prevent early entry into mitosis before cells are fully repaired [66]. Here, we show a new mechanism to ensure checkpoint maintenance by establishing Chk1 activation through Rad9A stabilization in case of genotoxic stress.

We also found that Rad9A and Chk1 coimmunoprecipitate in both directions in S-phase, which is reduced after prolonged exposure to BLEO or recovery from IR (Fig 6B and 6C). This coimmunoprecipitation in S-phase presents the possibility of Rad9A phosphorylation by Chk1, which could be part of a positive feedback loop to ensure Rad9A stabilization. It is interesting that Rad9A and Chk1 interaction in S-phase is reduced after prolonged exposure to BLEO. Chk1 cellular localization is required for checkpoint function, and exposure to DNA damage agents induces the release of phosphorylated Chk1 forms from chromatin to reach downstream targets such as Cyclin B/Cdk1 complex in the cytoplasm [68]. We have observed some level of activation of Chk1 with a single thymidine block when cells are synchronized in S-phase (Fig 6A). This agrees with previous research that report the activation of S-phase checkpoint with thymidine block because the inhibition of the elongation step of DNA replication [69]. An increased coimmunoprecipitation between Rad9A and Chk1 after a thymidine block suggests a model in which the activation of S-phase checkpoint induced by thymidine increases Rad9A and Chk1 interaction for Rad9A stabilization, and further damage produced by BLEO will increase the release of Chk1 from chromatin to reach cytoplasmatic targets to establish the checkpoint. These results present the possibility of an interaction between Rad9A and Chk1 that could be part of the checkpoint establishment.

We determined that Rad9A is degraded by an active system (Fig 7A and 7B) and inhibition of the proteasome system with MG132 increases Rad9A degradation (Fig 7A and 7B). Even more, we have found that prior treatment with MG132 reduces Rad9A hyperphosphorylation, Rad9A levels, and also, impairs checkpoint activation in cells exposed to BLEO (Fig 8). These results agree with previous studies that showed reduced Chk1 phosphorylation after IR exposure when the proteasome system is inhibited [56]. Since, we have determined that Rad9A accumulation after DNA damage is dependent on Chk1 activation (Figs 4 and 5) and previous treatment with MG132 affects Chk1 activation (Fig 8A), a reduction in Rad9A levels was expected. It was interesting to observe that proteasome inhibition with MG132 reduces Rad9A phosphorylation in cells exposed to BLEO (Fig 8A) while Chk1 inhibition with UCN-01 or siRNA increases Rad9A phosphorylation (Figs 4A and 5A). Chk1 inhibition with UCN-01 or Chk1 siRNA increases origin firing leading to an increased activity of ATR [47–49] and proteasome inhibition leads to reduced ATR activity [56]. An increased Rad9A phosphorylation in the case of Chk1 inhibition is explained by an increased ATR activation while a reduction of Rad9A phosphorylation with MG132 suggests a reduction in ATR activity. Proteasome inhibition with MG132 affects the IR-induce foci formation of phosphorylated ATM, 53BP1, NBS1, BRCA1, FANCD2, and RAD51 [56]. We observed a reduced BrdU incorporation with combined treatments of MG132 and BLEO (Fig 8C), and this could be explained by the effect of proteasome inhibition in IR-induce foci formation of Rad51 and for instance in the homologous recombination repair system. Our results showed that Rad9A is degraded in an active process that is different to the proteasome system.

Rad9A has been proposed to act as an oncogene [70,71]. Rad9A levels are increased in prostate [72] and breast cancer cells [73]. In fact, the ability of DU145 human prostate cancer cells to develop tumors into nude mice is eliminated with Rad9A knockdown [72], and the *in vitro* proliferation of MCF-7 breast cancer cells is inhibited with Rad9A silencing [73]. Our results suggest that a tight control of Rad9A levels is required to regulate the positive feedback loop between Rad9A and Chk1 in the DNA damage response; deregulation of this process could be responsible for the oncogenic activity of Rad9A described by others.

Some mechanistic aspects of the Rad9A-Chk1 interaction remain to be worked out in the future. Chk1 is known to phosphorylate and inactivate TLK1 kinase activity after DNA damage [74], and Kelly *et al*. (2013) showed that TLK1 phosphorylates Rad9A on threonine 355, and lack of this phosphorylation delays the progression through S-phase [75]. This opens the possibility that TLK1 links Chk1 and Rad9A in the process of checkpoint recovery [75]. Since, Chk1 activation prevents TLK1 kinase activity after DNA damage [74], and Chk1 activation is required for Rad9A stabilization (Figs 4 and 5), a possible explanation for Rad9A stabilization could be TLK1 inactivation by Chk1. Future work on Chk1-Rad9A positive feedback loop could focus on determining if Rad9A stabilization is mediated directly by Chk1 phosphorylation of Rad9A or indirectly by inhibiting TLK1 or another protein.
